# Numerical data for modelling pavement deflection behaviour under the TSD

**DOI:** 10.1016/j.dib.2024.111135

**Published:** 2024-11-15

**Authors:** Abdelgader Abdelmuhsen, Jean-Michel Simonin, Franziska Schmidt, Denis Lievre, Alexis Cothenet, Murilo Freitas, Amine Ihamouten

**Affiliations:** a Université Gustave Eiffel, MAST-LAMES, Nantes campus, F-44344 Bouguenais, France; b Université Gustave Eiffel, MAST-EMGCU, F-77454 Marne-la-Vallée Cedex 2, France

**Keywords:** Traffic speed deflectometer, Pavement mechanics, Subgrade resilient modulus, Numerical modeling, Alizé-Lcpc data, Road infrastructure assessment

## Abstract

This dataset provides a numerical simulation of pavement mechanical behavior under Traffic Speed Deflectometer (TSD) measurements. It consists of simulated deflection slope data for various pavement structures and subgrade properties, generated using the Alizé-LCPC software, a standard tool in French pavement engineering. The dataset addresses limitations in traditional Falling Weight Deflectometer (FWD) methods, offering a more accurate and computationally efficient approach for estimating the Subgrade Resilient Modulus (M_R_) using machine learning models. This resource is valuable for researchers aiming to enhance pavement evaluation methods and develop predictive models for road infrastructure maintenance and assessment. The data are openly accessible, facilitating widespread research collaboration and the application of advanced data analytics in pavement engineering.

Specifications Table

**Table:** 

Subject	Civil and Structural Engineering.
Specific subject area	Pavement Mechanics and Numerical Simulation Data*.*
Type of data	Table, Image, Dataset, Graph, Processed.
Data collection	A synthetic dataset was created using Alizé-LCPC, integrating multiple material and structural parameters. It included 235 distinct instances representing various combinations of subgrade properties*.*
Data source location	Univ Gustave *Eiffel, Nantes* campus, France, 2022*.*
Data accessibility	1. Publicly available in a data repository: ABDELGADER, Abdelmuhsen, SIMONIN, Jean-Michel, SCHMIDT, Franziska, LIEVRE, Denis, COTHENET, Alexis, FREITAS, Murilo, & IHAMOUTEN, Amine. (2024). “Numerical modeling of pavement deflection behavior under The Traffic Speed Deflectometer.” Recherche Data Gouv. DOI: 10.57745/QNUCI6. UNF:6 + 1mG1 × 7Ts/x/t0doInQTw==. Version V1.
	Repository name: *Recherche Data Gouv*Data identification number: 10.57745/QNUCI6. Direct URL to data: Numerical modeling of pavement deflection behavior under The Traffic Speed Deflectometer - Data Univ. Gustave EiffelIPublicly available in a data repository
Related research article	Abdelmuhsen, A., Simonin, J.-M., Schmidt, F., Lievre, D., Cothenet, A., & Ihamouten, A. (2023). "On the variants of SVM method for the estimation of soil elastic modulus from TSD model: Numerical parametric study." Transportation Engineering, 13, 100,187. 10.1016/j.treng.2023.100187I

## 1 Value of the Data

• Access to Proprietary Data: Generated using the industry-standard Alizé-LCPC software, this dataset provides high-quality pavement modeling data, typically inaccessible due to the softwareʼs high cost and restricted access. By making this resource publicly available, it offers researchers access to state-of-the-art data that would otherwise be difficult to obtain, broadening opportunities for high-impact studies in pavement engineering.
• Enabling Advanced Research: The dataset facilitates the use of advanced machine learning models to estimate the Subgrade Resilient Modulus (M_R_), a critical factor in pavement performance. It allows researchers to develop innovative methodologies that go beyond traditional FWD techniques, improving the precision and efficiency of pavement assessments.
• Foundation for Future Research: As a rare, publicly available resource, this dataset serves as a reference for future pavement studies. It supports the comparison of different evaluation models and methods, helping to refine and standardize practices in pavement performance research and enhancing the accuracy of predictive models.
• Advancing Pavement Engineering: The dataset's comprehensive TSD-based deflection slope measurements are essential for developing more accurate predictive models of pavement foundation health. It supports advancements in methodologies for pavement performance evaluation and structural health monitoring, contributing to improved road infrastructure management.

## 2 Background

The dataset was developed to overcome challenges associated with traditional pavement evaluation methods, particularly those using the Falling Weight Deflectometer (FWD). FWD is widely used for estimating the Subgrade Resilient Modulus (M_R_) of pavements, a key parameter for assessing the structural health and performance of road foundations. However, FWD methods have several drawbacks, including safety concerns during field measurements. These issues limit the applicability and efficiency of FWD in large-scale pavement management system. To address these limitations, this dataset utilizes Traffic Speed Deflectometer (TSD) simulated measurements, which provide continuous and non-intrusive assessment of pavement. By integrating TSD data with machine learning techniques, the dataset supports the development of an inverse model that accurately estimates M_R_ from TSD deflection slope measurements. The dataset is generated using Alizé-LCPC, a proprietary software tool that has been the standard for pavement design and analysis in France for over three decades. The data includes a range of simulated pavement behaviors under varying conditions, making it a valuable for researchers working to improve pavement evaluation methodologies to predict models for infrastructure management [[1], [2], [3], [4], [5], [6], [7], [8]].

## 3 Data Description

**Fig. 1:**
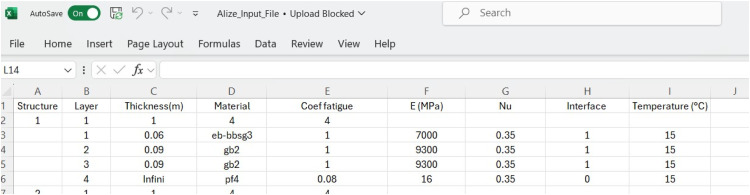
Alizé-Lcpc Output Files.
  Simulated TSD Deflection Slope Files: This dataset consists of 235 files, each corresponding to deflection slope data for specific subgrade modulus values under dual-wheel loading. These files capture the impact of varying subgrade stiffness on pavement behavior. Generated by Alizé-LCPC, each file represents pavement deflection for a unique soil modulus. The data are then combined into a single file, with each column representing a distinct modulus value. Below is a Fig. 2, for the Alizé-LCPC output files.

The dataset consists of the following components, each structured to provide detailed insights into pavement behavior under TSD loading conditions:

1. Alizé-LCPC Input File: This file includes essential configuration parameters for numerical simulation, such as TSD load settings, pavement structure details, and subgrade properties. It serves as the foundational input for generating simulated deflection slopes and comprises 235 distinct soil modulus values to model deflection behavior under various soil conditions. The data processing workflow begins with this file, which is crucial for defining the parameters of the target pavement structure. Below is a Fig. 1, for the Alizé-LCPC input file.

**Fig. 2:**
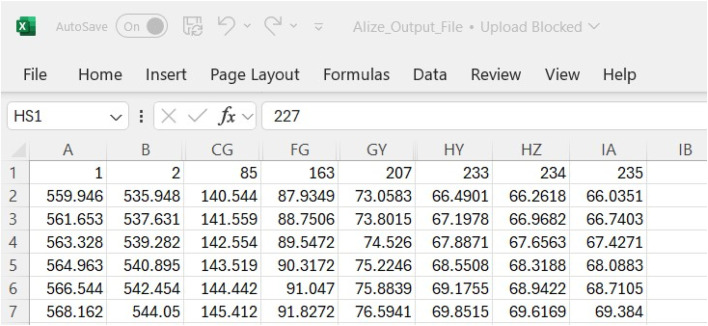
Alizé-Lcpc Output Files.
2. Processed Slope Simulation Data: This file compiles deflection slopes from Alizé-LCPC outputs, offering corrected values and detailed metrics, such as horizontal offset, total deflection, and contributions from individual wheels. It contains data for 235 soil modulus values simulated through a Python-based program using the superposition principle and first derivative method. The columns represent variables like horizontal offset from a 3.8-meter reference, total deflection under the TSD's rear wheels (µm), and sensor distances. It also includes individual wheel deflections, aggregated deflection, and the rate of slope change per unit length (µm/m). Corrected slope values enhance accuracy at the 3.8-meter mark. Below is a Fig. 3, for the Python simulation file.

**Fig. 3:**
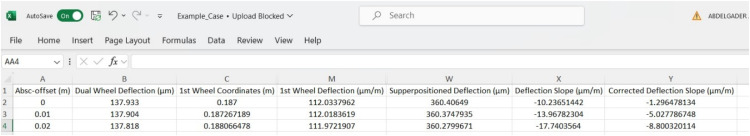
Python Slope Simulation File.
3. Concatenated Deflection Slope Database: This dataset integrates all 235 simulated deflection slope files into a single, unified database. It enables a comprehensive analysis of how subgrade properties affect pavement deflection responses. The file consolidates deflection data for all 10 TSD wheels, computed using the superposition principle and first derivative method. This combined dataset is particularly valuable for training and validating machine learning models. Below is a Fig. 4, for the concatenated deflection slope database file.

**Fig. 4:**
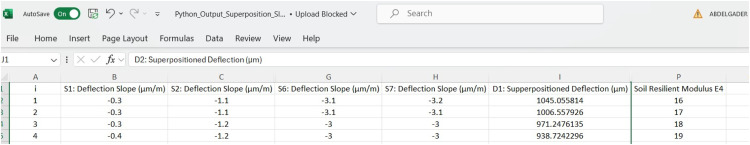
Python Slope Simulation File.

## 4 Experimental Design, Materials and Methods

**Numerical Forward Modelling:**

The design of this dataset involves a detailed numerical modeling approach to simulate the mechanical behavior of pavement structures under traffic loads using the Alizé-LCPC software. This section outlines the key components and methodologies employed in constructing the dataset, which serves as a foundation for evaluating pavement performance and supports the development of machine learning models for predicting the Subgrade Resilient Modulus (M_R_). The structured methodology presented here ensures that the dataset is both robust and applicable for a wide range of research applications in pavement engineering.

1. Alizé-LCPC Software

Alizé-LCPC, a proprietary tool widely used for pavement design in France, was employed to develop the numerical forward model. This software integrates Burmister's multi-layer elastic theory, enabling accurate computation of pavement responses, such as stresses, strains, and deflections, under various loading conditions. The model adheres to French standards (NF P98–086/2019), which set strict criteria for material fatigue and rutting behavior, ensuring the integrity and reliability of the simulated data. The use of Alizé-LCPC allows for precise calibration of material properties and structural parameters, which are crucial for generating high-fidelity simulations of pavement behavior under traffic loads.

2. Database Parameters

The dataset construction involved defining two primary components: the pavement structure and the Traffic Speed Deflectometer (TSD) load configuration. The characterization of these parameters is critical to capturing realistic pavement responses and ensuring the validity of the simulated data.

3. Pavement Structure: Mechanical Properties

The mechanical properties of the pavement layers were meticulously defined to reflect typical road structures used in France. The key parameters include:

• Layer Thickness (m): Specifies the depth of each pavement layer, influencing load distribution and deformation.
• Material Type: Includes various materials such as Hot Mix Asphalt (HMA) and Bituminous Concrete (BC-g2), each contributing differently to the pavement's mechanical performance.
• Elastic Modulus (E_i_): Denotes the stiffness of each layer in megapascals (MPa), which is crucial for assessing the structural capacity and load-bearing behavior.
• Poisson's Ratio (Nu): Describes the material's lateral deformation in response to axial stress, affecting overall pavement deflection characteristics.
• Interface Condition: Defines the bonding state between layers (e.g., bonded or unbonded), which influences load transfer and structural integrity.
• Temperature (°C): Material properties were specified at a constant temperature of 15 °C to minimize the effects of thermal variations on the simulation results.

**Fig. 5:**
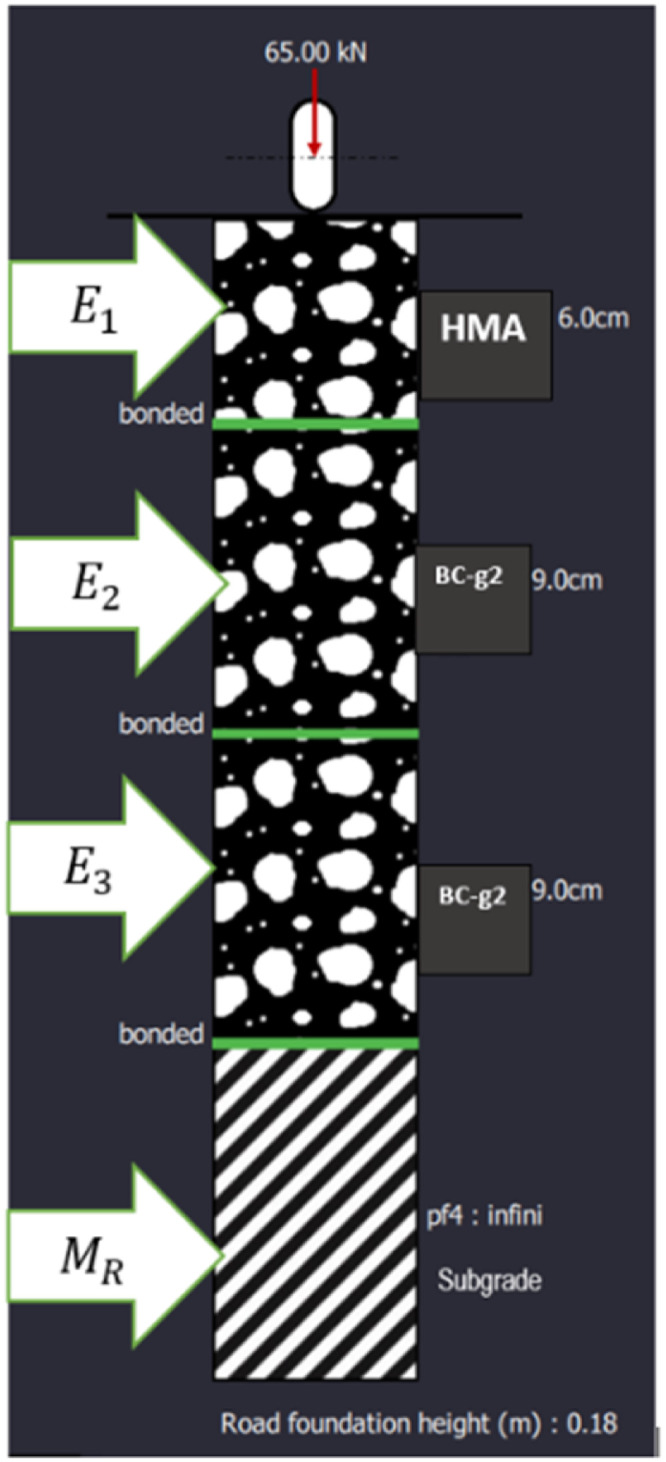
Structure of the pavement.

As shown in Table 1 and Fig. 5, the primary focus was on accurately estimating the resilient modulus (M_R_) of the subgrade, a key indicator of pavement performance. The forward model simulated deflection slopes (S_D_) across a range of M_R_ values, providing a comprehensive dataset for training machine learning models. This synthetic dataset was rigorously constructed through predefined parameters and feature engineering to ensure precise analysis and categorization of M_R_ values, facilitating reliable prediction and validation of pavement behavior.

**Table 1:** The characteristics of the pavement structure utilized to derive the forward model from the French catalog.

Layer (i)	Thickness (m)	Material	E_i_ (MPa)	v	Interface	°C
1	0.06	HMA	7000	0.35	Bound	15
2	0.09	BC-g2	9300	0.35	Bound	15
3	0.09	BC-g2	9300	0.35	Bound	15
4	∞	M_R_ (Pf_4_)	16–250	0.35	Bound	15

In scientific terminology, HMA denotes Hot Mix Asphalt, BC-g2 represents grade 2 aggregate bituminous concrete, and pf_4 refers to class 4 subgrade.

### 4.1 Traffic speed deflectometer (TSD) configuration

The TSD is a cutting-edge device used for continuous, high-speed measurement of pavement deflections, offering a non-intrusive method for evaluating pavement structural health. The dataset includes detailed simulations of the TSD's operational characteristics, essential for accurately capturing pavement responses under real-world traffic conditions.

### 4.2 TSD measurement coordinates

The spatial configuration of the TSD measurements is detailed in Table 2, which includes:

• Vertical Distance (R_x_): The vertical offset between the load reference point and the sensor, critical for accurately measuring pavement deflection profiles.
• Horizontal Distance (R_y_): The lateral distance from the load application point to the sensor, essential for capturing the full extent of the deflection basin.
• Load per Wheel (F): The load exerted by each wheel, measured in tons, affecting the stress distribution and resulting pavement deflections.

**Table 2:** Coordinates of TSD simulated measurements with load values per wheel.

Load	L_1_	L_2_	L_3_	L_4_	L_5_	L_6_	L_7_	L_8_	L_9_	L_10_
R_x_ (m)	0	0	0	0	08.150	8.150	8.150	8.150	11.75	11.75
R_y_ (m)	−0.187	−0.187	1.913	2.287	−0.187	0.187	1.913	2.287	−0.187	2.287
F (ton)	2.875	2.875	2.875	2.875	1.55	1.55	1.55	1.55	3.15	3.15

### 4.3 TSD load configuration

Table 3 outlines the TSD's loading parameters, crucial for simulating realistic pavement responses:

• Axle Load (F): The total force applied by the TSD axle in kilonewtons (kN), directly influencing the magnitude of deflections.
• Contact Pressure (P): The tire-pavement contact pressure in megapascals (MPa), which determines localized stress distribution.
• Speed (V_h_): The constant velocity of the TSD vehicle in meters per second (m/s), affecting the dynamic response of the pavement.
• Tire Radius (R): The radius of the TSD tires, influencing the contact area and load application.
• Load Frequency (f): The frequency of applied load cycles in Hertz (Hz), relevant for analyzing pavement fatigue behavior under repetitive loading.

**Table 3:** Coordinates of TSD simulated measurements with load values per wheel.

Parameter	F (KN)	P (MPa)	V_h (m/s)_	R (m)	F (Hz)
Value	32.5	0.92	20	0.15	10

### 4.4 Laser Doppler sensor position

The positioning of Laser Doppler sensors relative to the TSD load, as detailed in Table 4, is critical for capturing precise deflection data:

• Sensor Position (S_x_): The horizontal distance from the load application point to each sensor, measured in meters. Accurate sensor positioning is necessary to detect and analyze subtle variations in pavement deflection behavior.

**Table 4:** TSD Laser Doppler sensor position.

Sensor	S_1_	S_2_	S_3_	S_4_	S_5_	S_6_	S_7_	S_8_
(S_x_) (m)	0.1	0.2	0.3	0.45	0.6	0.9	1.1	3.8

This setup, integrating detailed material properties and precise TSD load configurations, underpins the generation of a high-quality dataset. The dataset enables comprehensive evaluation and modeling of pavement behavior, providing a robust platform for advancing research in pavement engineering. By making this data publicly accessible, we aim to facilitate its use in the development of innovative predictive models and support data-driven decision-making in road infrastructure management [[1], [2]]*.*

## Limitations

The dataset, while comprehensive in its scope, is based on numerical simulations and may not fully capture the complexities of in-situ pavement behaviour.

## Ethics Statement

The authors confirm that they have adhered to the ethical guidelines for publication in Data in Brief. This paper does not involve human subjects, animal experiments, or data derived from social media platforms*.*

## Credit Author Statement

**A. Abdelmuhsen:** Conceptualization, Methodology, Data Curation, Writing - Original Draft, **Jean-M. Simonin:** Supervision, Validation, Writing - Review & Editing, **F. Schmidt:** Software, Data Processing, Visualization, **D. Lièvre:** Resources, Data Curation, Methodology, **A. Cothenet:** Investigation, Validation, Data Analysis, **M. Freitas:** Formal Analysis, Writing - Review & Editing, **A. Ihamouten:** Project Administration.

## Data Availability

License : etalab 2.0Numerical modeling of pavement deflection behavior under The Traffic Speed Deflectometer (Original data).
